# The clinical diversity of primary hypothyroidism presenting as a spontaneous ovarian hyperstimulation syndrome

**DOI:** 10.1530/EDM-23-0084

**Published:** 2024-09-27

**Authors:** Krishna Prabha, K Felix Jebasingh, Vaibhav Londhe, Nihal Thomas

**Affiliations:** 1 Department of Endocrinology, Diabetes and Metabolism, Christian Medical College, Vellore, Tamil Nadu, India; 2 Department of Obstetrics and Gynaecology, Unit II, Christian Medical College and Hospital, Vellore, Tamil Nadu, India

## Abstract

**Summary:**

Ovarian hyperstimulation syndrome (OHSS) usually occurs in patients undergoing assisted reproduction techniques and ovulation induction. Its variant, spontaneous ovarian hyperstimulation syndrome, a potentially life-threatening disorder, is uncommon and only a few cases have been reported in association with hypothyroidism. This study analysed five patients with untreated chronic hypothyroidism presenting with multicystic ovaries, isosexual precocious puberty, and delayed bone age; subsequently, the follow-up and regression of ovarian pathology was assessed. Two patients had presented to the emergency department with menorrhagia and hypotension, of these, one had ovarian torsion at presentation. Three patients presented to the outpatient department: one for evaluation of short stature, one for premature menarche, and another with polycystic ovaries. They were all diagnosed with long-standing, untreated chronic hypothyroidism. There was regression of the size of the cystic ovaries on subsequent follow-up. In all these patients, long-standing hypothyroidism had resulted in ovarian hyperstimulation syndrome. The potentially life-threatening complications of this syndrome may be prevented by careful screening and a strong index of clinical suspicion at the outset.

**Learning points:**

- Long-standing, untreated primary hypothyroidism may result in spontaneous ovarian hyperstimulation syndrome.
- A high index of suspicion is required for an early and accurate diagnosis.
- The requirement for interdepartmental collaboration between gynaecology and endocrinology departments is essential for the successful management of this life-threatening but easily treatable disorder.

## Background

The incidence of ovarian hyperstimulation syndrome (OHSS) in women undergoing ovulation induction therapy for *in vitro* fertilisation is 0.2–1%. It is seldom seen in patients who have not undergone medical ovulation induction (1). OHSS is characterised by bilateral symmetrical enlargement of the ovaries with multiple cysts, accompanied by ascites, pleural effusions, and pericardial effusion. This case series highlights the initial presentation of young ladies with long untreated hypothyroidism as medical emergencies requiring management in intensive care units and who were suspected to have ovarian malignancies before definitive diagnosis (2, 3).

There have been a number of case reports involving spontaneous OHSS in association with pregnancy and a few case reports of spontaneous OHSS with pregnancy and untreated hypothyroidism (4, 5). Ovarian hyperstimulation syndrome in two members of a family with autoimmune hypothyroidism was described by Hedayati *et al.* (6). There are very few case reports of ovarian hyperstimulation syndrome in untreated primary hypothyroidism. Moreover, documentation of regression of the ovarian cysts with successful treatment is sparse.

## Case presentation

### Patient 1 (P1)

**Figure 1:**
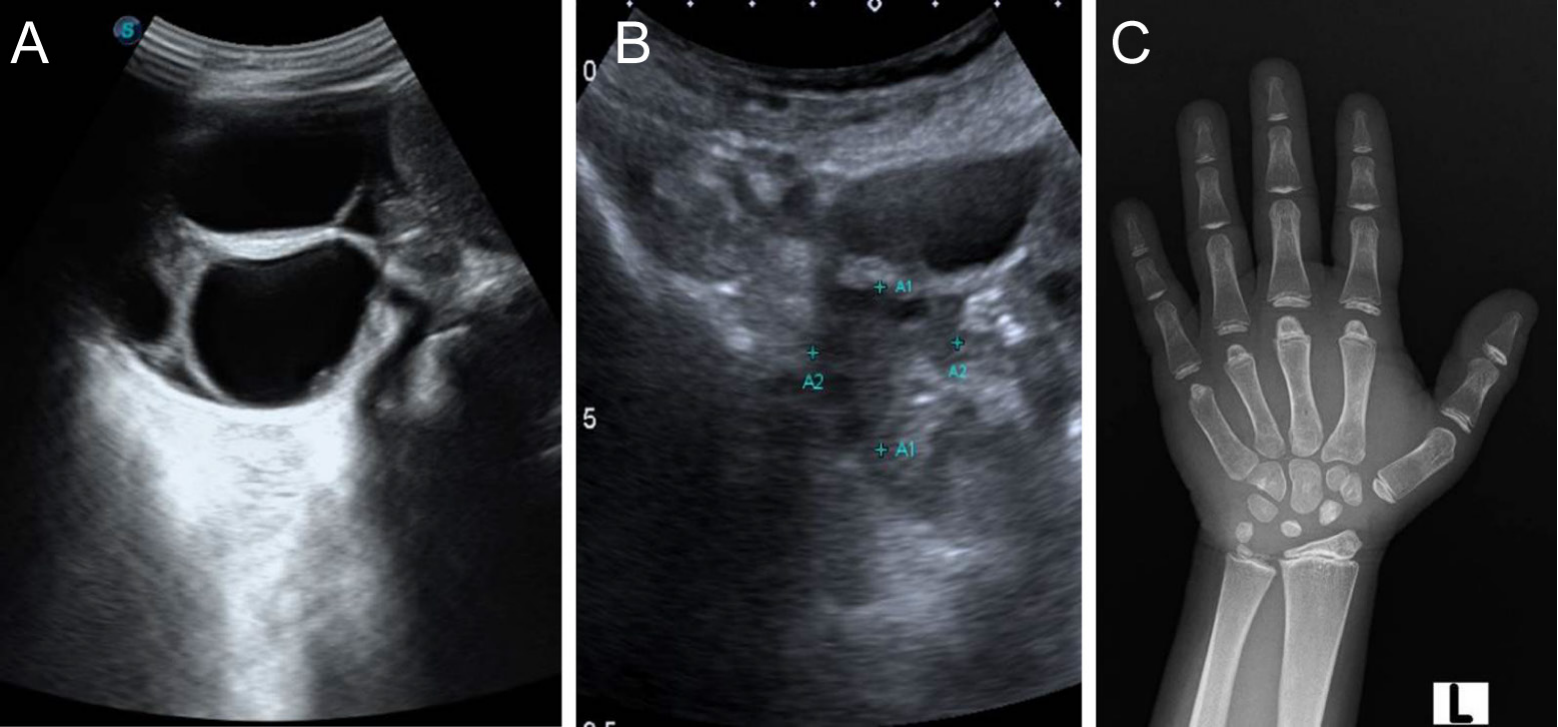
(A) Ultrasound of the abdomen at presentation with multiple cysts and ovarian volume of 50 mL. (B) Ultrasound abdomen at follow-up with resolved cyst. (C) P1 presented at 21 years with a delayed bone age of 9 years.

A 22-year-old lady was brought to the emergency department (ED) with acute abdominal pain. She had vomiting and abdominal distension over 4 days. An emergency computed tomography (CT) of the abdomen revealed left ovarian torsion. She underwent a diagnostic laparoscopy and left side salpingo-oohrectomy. She was the youngest of three siblings of a fourth-degree consanguineous marriage. Her mother’s antenatal and intrapartum history was uneventful; however, she had delayed milestones and evolution of speech was delayed. Her mother had observed that she was of short stature, but the child had never been evaluated. She had attained menarche at 14 years, and her cycles were infrequent with associated heavy menstrual bleeding. On examination, her height was 115 cm, (<3rd centile) with a height score −2 SDS. She had dysmorphic features: low-set ears and a depressed nasal bridge. Sexual maturity with Tanner staging showed P1B4 (absent pubic hair). Her TSH was 652.16 mIU/L with a serum free T4 of 1.8 pmol/L (Tables 1 and 2). years. Her ultrasound abdomen revealed large multicystic ovaries which resolved with treatment (Fig. 1A and B). Radiology of the left wrist showed a delayed bone age of 9 years (Fig. 1C). She was started on oral levothyroxine 12.5 mcg once daily which was increased to 75 mcg once daily, and she is currently under follow-up (Table 2). The ultrasound of the neck revealed thyroid agenesis. She is asymptomatic at present with resolution of ovarian cysts.

**Table 1:** Clinical course of patients P1-P5 (A to E) - age at presentation and clinical features.

Parameter	P1	P2	P3	P4	P5
Age (current)	22	18	26		22
Age (at presentation)	21	16	7	17	21
Tanner at presentation	B5P1	B4P1	B3P1	B3P1	B4P1
Presentation	Left ovarian torsion	Ovarian mass	Early menarche	polyserositis	Ovarian mass/serositis
Thyroid stimulating hormone (mIU/L)	652.526	750	1600	750	595
Ultrasonogram thyroid	Thyroid agenesis	Cystic nodules thyroid	Dysplastic thyroid		

**Table 2:** Baseline characteristics of patients (P1 - P5) with biochemical parameters.

Parameter	P1	P2	P3	P4	P5	Mean ± s.d.	Reference ranges
Hemoglobin (gm%)	1.6	7.8	10.6	2.4	10.5	6.58 ± 4.3	11–15
Follicle stimulating hormone (IU/L)	2.72		6.8	7.9		5.8 ± 2.7	2.8–11.3
Luteinizing hormone (IU/L)	4.08		8.1	0.5		4.2 ± 3.2	1.1–11.6
Prolactin in dilution (mIU/L)	987.2		189.3	848.9		675.1 ± 348	59.57–621.2
Thyroid stimulating hormone (mIU/L)	652.56	750	1600	750	595	869 ± 413	0.5–4.78
Total thyroid hormone T4 (nmol/L)	6.43	109.4	32.18	3.86	1.28	30.6 ± 40	57.9–140.3
Free thyroid hormone, FT4 (pmol/L)	1.8	7.7	2.83	2.57	0.51	3.08 ± 2.4	10.2–25.7

### Patient 2 (P2)

**Figure 2:**
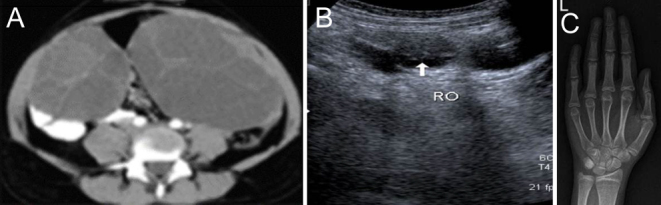
(A) CT abdomen at presentation with large multilocular cysts probable mucinous cystadenoma. (B) Ultrasound abdomen at follow-up with resolved cyst. (C) P2 presented at 16 years with delayed bone age of 9 years..

A 16-year-old girl was referred from Gynaecology for evaluation of bilateral polycystic ovaries. She was referred from another institution with the suspicion of ovarian malignancy (Fig. 2A). She had been previously diagnosed with primary hypothyroidism at the age of 10 years, and her initial TSH was >100 mIU/L (Tables 1 and 2). However, she had been noncompliant with medications. Her height was 143 cm, which was less than the 3rd centile (SDS-3), and her bone age of the left wrist was 9 years (Fig. 2C). She had attained menarche at 13 years of age and had regular menstrual cycles. Her Tanner staging was B4P1. Her TSH was 750 mIU/L with a free T4 of 7.7 pmol/L. She was started on oral levothyroxine 50 mcg, which was increased to 100 mcg once daily and at 2 years of follow-up, her ovarian cysts had resolved (Fig. 2B).

### Patient 3 (P3)

**Figure 3:**
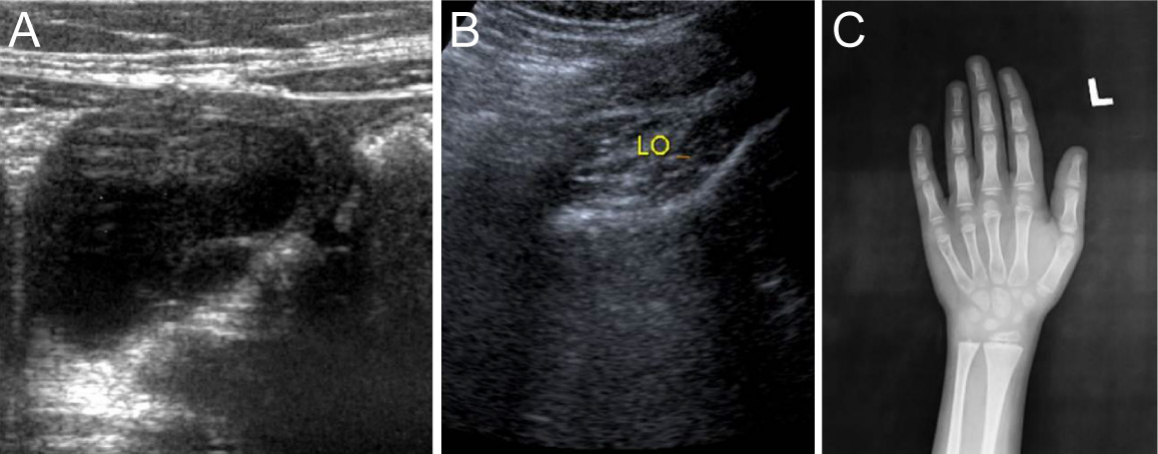
(A) Ultrasound abdomen at presentation with polycystic ovaries. (B) Ultrasound abdomen at follow-up with resolved cyst; she conceived and had two children. (C) P3 presented at 7 years with premature menarche but delayed bone age less than 6 years.

A 7-year-old girl presented with premature menarche. On examination, her height was less than the third centile for age and Tanner's stage was B3P1. Her abdominal ultrasound showed large multicystic ovaries (Fig. 3A). Her TSH was 1600 mIU/L with a low free T4 of 2.8 pmol/L. She was started on oral levothyroxine 150 mcg once daily (Tables 1 and 2). She was followed up annually, the ovarian cysts had regressed (Fig. 3B). Her bone age was 6 years at presentation (Fig. 3C). Subsequently, there was a gradual increase in height, and at present, at 27 years of age, she has a height of 158 cm (25th centile), is married, and has two children.

### Patient 4 (P4)

A 17-year-old girl presented to the gynaecology emergency with heavy and prolonged menstrual cycles and was in hypovolemic shock. Her haemoglobin was 3 gm%. She was resuscitated and required care in the intensive unit with multiple packed cell transfusions. On examination, she had a height less than the third centile and a Tanner’s stage of B3P1 (Tables 1 and 2). She had a delayed bone age of 11 years. Her emergency ultrasound of the abdomen showed large multicystic ovaries in addition to ascites, pleural, and pericardial effusion. Her TSH was 750 mIU/L and free T4 was 2.57 pmol/L. She was started on levothyroxine 75 mcg once daily and there was resolution of cysts on follow-up.

### Patient 5 (P5)

**Figure 4:**
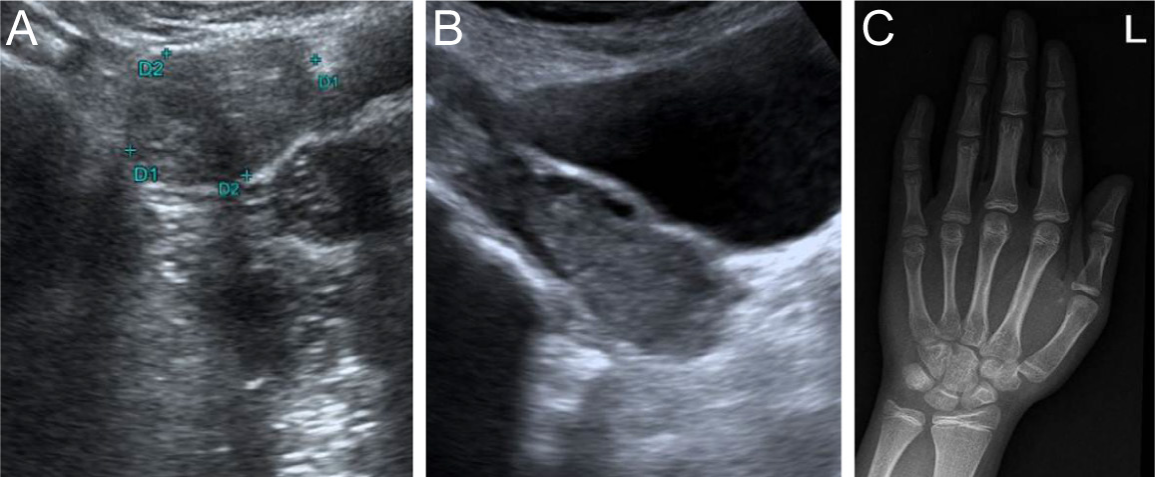
(A) Ultrasound abdomen at presentation with multicystic ovaries with ovarian volume 30 mL. (B) Ultrasound abdomen at follow-up with resolved cyst. (C) P5 presented at 22 yrs with polyserositis and delayed bone age of 11 years.

A 22-year-old woman presented to the outpatient department with abdominal pain, exertional dyspnoea, and episodes of menorrhagia. On examination, she had short stature less than the third percentile for age (144 cm), and a Tanner staging of B4P1. The ultrasound of the abdomen showed enlarged multicystic ovaries (Fig. 4A). She had a delayed bone age of 11 years (Fig. 4C). The evaluation revealed the presence of pleural and pericardial effusions. The TSH was 595 mIU/L and the free T4 was 0.51 pmol/L. She was started on levothyroxine supplements at 125 mcg once daily, and there was resolution of ovarian cysts at 1 year follow-up.(Tables 1 and 2) (Fig. 4B). All five patients had short stature and delayed bone age at presentation. Thyroid antibodies (antithyroid peroxidase antibodies) were negative. The aetiology of long-standing hypothyroidism included thyroid dysgenesis and the presence of a dysplastic thyroid.

## Investigation

Details are presented in Table 2.

## Treatment

All five patients were initiated on tab levothyroxine at doses up to 1.6 mcg/kg per oral once daily and followed up with dose titration.

## Outcome and follow-up

All patients were followed up for a period of 2–5 years. There was resolution of ovarian cysts on follow-up in all the patients.

## Discussion

Spontaneous ovarian hyperstimulation syndrome occurring in patients with untreated hypothyroidism is a rare occurrence, and very few cases have been reported in the literature. The risk factors for OHSS include youth, an asthenic habitus, luteal hCG supplementation, GnRH protocols, increased serum oestradiol, and the presence of multiple follicles (7). The syndrome has been reported between 8 and 14 weeks of pregnancy and in the presence of the follicle-stimulating hormone (FSH) secreting pituitary adenoma (8). Other uncommon causes include multiple pregnancies with increased hCG, hydatidiform moles, and raised TSH as in untreated hypothyroidism (8). TSH, FSH, LH, and HCG belong to a family called glycoprotein hormones. The receptors for these hormones share a similar structure. Normally, hCG and LH bind to the LH receptor, while FSH and TSH bind to separate FSH and TSH receptors, respectively. Thus, it involves the presence of downstream signaling activation. De Leener *et al*. classified spontaneous ovarian hyperstimulation syndrome into three types based on the clinical features and FSH receptor mutation:

- Type I: corresponds to mutated FSH receptor cases
- Type II: corresponds to spontaneous OHSS and a high hCG. This is the most common.
- Type III: related to hypothyroidism (9).

All the above patients had type III OHSS.

The mechanism of OHSS in hypothyroidism is poorly understood. A possible explanation was proposed by Rotmensch and Scommegna (10), on the basis of preferential formation of estriol via the 16-hydroxylation pathway instead of the normal 2-hydroxylation that has been demonstrated in patients with hypothyroidism. Excessive release of gonadotropins, due to the decreased feedback regulation caused by the substitution of oestradiol by the less potent oestriol would result in excessive ovarian stimulation (5, 10). The strength of our study involved the prompt collaborative effort between several specialities in the diagnosis of these patients. The limitation involves the lack of sonology of the thyroid in two patients. Appropriate and expeditious identification of the aetiology is of crucial importance in this potentially life-threatening disorder of the ovarian hyperstimulation syndrome. There is no definitive therapy, other than supportive care and treatment of the underlying cause in these patients. This case series highlights the variability of presentation of untreated hypothyroidism and the high index of clinical suspicion that may be required by the treating physician to establish a diagnosis.

## Declaration of interest

The authors declare that there is no conflict of interest that could be perceived as prejudicing the impartiality of the study reported.

## Funding

This research did not receive any specific grant from any funding agency in the public, commercial, or not-for-profit sector.

## Patient consent

Informed consent was not obtained from the participants, and waiver of consent was obtained from the institutional review board (since there is no intervention). The IRB clearance number is 15278, dated 22 March 2023.

## Author contribution statement

Dr KP - involved in the preparation of the manuscript. Dr FJ - research and study design and data collection. Dr VL - review of manuscript and revision of data. Dr NT - research, data collection, interpretation, guidance and support.
